# Supplementary material for: Development of a computer-aided design software for the quantitative evaluation of aesthetic damage
Source: PLoS One. 2019 Dec 18;14(12):e0226322. doi: 10.1371/journal.pone.0226322 (PMC6919621; doi:10.1371/journal.pone.0226322)
Supplement: S1 Code site — 1. (ZIP) [file pone.0226322.s001.zip › [EESC_jr][Projeto]Relatorio_07_21/reports/templates_zip/gentelella-master.zip!/gentelella-master/vendors/DateJS/reports/lcov-report/core/parser.js.html]

Code coverage report for core/parser.js


# Code coverage report for core/parser.js

## Statements: 72.34% (34 / 47)      Branches: 87.5% (28 / 32)      Functions: 62.5% (5 / 8)      Lines: 72.34% (34 / 47)      Ignored: none

All files » core/ » parser.js

```
|  |  |  |
| --- | --- | --- |
| 1 2 3 4 5 6 7 8 9 10 11 12 13 14 15 16 17 18 19 20 21 22 23 24 25 26 27 28 29 30 31 32 33 34 35 36 37 38 39 40 41 42 43 44 45 46 47 48 49 50 51 52 53 54 55 56 57 58 59 60 61 62 63 64 65 66 67 68 69 70 71 72 73 74 75 76 77 78 79 80 81 82 83 84 85 86 87 88 89 90 91 92 93 94 95 96 97 98 99 100 101 102 103 104 105 106 107 108 109 110 111 112 113 114 115 116 117 118 119 120 121 122 123 124 125 126 127 128 129 130 131 132 133 134 135 136 137 138 139 140 141 142 143 144 145 146 147 148 149 150 151 152 153 154 155 156 157 158 159 160 161 162 163 164 165 166 167 168 169 170 171 172 173 174 175 176 177 178 179 180 181 182 183 184 185 186 187 188 189 190 191 192 193 194 195 196 197 198 199 200 201 202 203 204 205 206 207 | 1 1                                                                                                                                                                                                           1   530 530 530     530 530 530         530     2 2   2 2           1 620 620 6   614 3   611   449   611 81     530 530 530 528   2         1 1   1   1                                                                                 1 | ``` (function () { 	var $D = Date;   	/** 	 * @desc Converts the specified string value into its JavaScript Date equivalent using CultureInfo specific format information. 	 *  	 * Example 	<pre><code> 	/////////// 	// Dates // 	///////////   	// 15-Oct-2004 	var d1 = Date.parse("10/15/2004");   	// 15-Oct-2004 	var d1 = Date.parse("15-Oct-2004");   	// 15-Oct-2004 	var d1 = Date.parse("2004.10.15");   	//Fri Oct 15, 2004 	var d1 = Date.parse("Fri Oct 15, 2004");   	/////////// 	// Times // 	///////////   	// Today at 10 PM. 	var d1 = Date.parse("10 PM");   	// Today at 10:30 PM. 	var d1 = Date.parse("10:30 P.M.");   	// Today at 6 AM. 	var d1 = Date.parse("06am");   	///////////////////// 	// Dates and Times // 	/////////////////////   	// 8-July-2004 @ 10:30 PM 	var d1 = Date.parse("July 8th, 2004, 10:30 PM");   	// 1-July-2004 @ 10:30 PM 	var d1 = Date.parse("2004-07-01T22:30:00");   	//////////////////// 	// Relative Dates // 	////////////////////   	// Returns today's date. The string "today" is culture specific. 	var d1 = Date.parse("today");   	// Returns yesterday's date. The string "yesterday" is culture specific. 	var d1 = Date.parse("yesterday");   	// Returns the date of the next thursday. 	var d1 = Date.parse("Next thursday");   	// Returns the date of the most previous monday. 	var d1 = Date.parse("last monday");   	// Returns today's day + one year. 	var d1 = Date.parse("next year");   	/////////////// 	// Date Math // 	///////////////   	// Today + 2 days 	var d1 = Date.parse("t+2");   	// Today + 2 days 	var d1 = Date.parse("today + 2 days");   	// Today + 3 months 	var d1 = Date.parse("t+3m");   	// Today - 1 year 	var d1 = Date.parse("today - 1 year");   	// Today - 1 year 	var d1 = Date.parse("t-1y");      	///////////////////////////// 	// Partial Dates and Times // 	/////////////////////////////   	// July 15th of this year. 	var d1 = Date.parse("July 15");   	// 15th day of current day and year. 	var d1 = Date.parse("15");   	// July 1st of current year at 10pm. 	var d1 = Date.parse("7/1 10pm"); 	</code></pre> 	 * 	 * @param {String}   The string value to convert into a Date object [Required] 	 * @return {Date}    A Date object or null if the string cannot be converted into a Date. 	 */ 	var parseUtils = { 		removeOrds: function (s) { 			ords = s.match(/\b(\d+)(?:st\|nd\|rd\|th)\b/); // find ordinal matches 			s = ((ords && ords.length === 2) ? s.replace(ords[0], ords[1]) : s); 			return s; 		}, 		grammarParser: function (s) { 			var r = null; 			try { 				r = $D.Grammar.start.call({}, s.replace(/^\s*(\S*(\s+\S+)*)\s*$/, "$1")); 			} catch (e) { 				return null; 			} 			 			return ((r[1].length === 0) ? r[0] : null); 		}, 		nativeFallback: function(s) { 			var t; 			try { 				// ok we haven't parsed it, last ditch attempt with the built-in parser. 				t = Date._parse(s); 				return (t \|\| t === 0) ? new Date(t) : null; 			} catch (e) { 				return null; 			} 		} 	}; 	function parse (s) { 		var d; 		if (!s) { 			return null; 		} 		if (s instanceof Date) { 			return s.clone(); 		} 		if (s.length >= 4 && s.charAt(0) !== "0" && s.charAt(0) !== "+"&& s.charAt(0) !== "-") { // ie: 2004 will pass, 0800 won't. 			//  Start with specific formats 			d = $D.Parsing.ISO.parse(s) \|\| $D.Parsing.Numeric.parse(s); 		} 		if (d instanceof Date && !isNaN(d.getTime())) { 			return d; 		} else { 			// find ordinal dates (1st, 3rd, 8th, etc and remove them as they cause parsing issues) 			s = $D.Parsing.Normalizer.parse(parseUtils.removeOrds(s)); 			d = parseUtils.grammarParser(s); 			if (d !== null) { 				return d; 			} else { 				return parseUtils.nativeFallback(s); 			} 		} 	}   	Eif (!$D._parse) { 		$D._parse = $D.parse; 	} 	$D.parse = parse;   	Date.getParseFunction = function (fx) { 		var fns = Date.Grammar.allformats(fx); 		return function (s) { 			var r = null; 			for (var i = 0; i < fns.length; i++) { 				try { 					r = fns[i].call({}, s); 				} catch (e) { 					continue; 				} 				if (r[1].length === 0) { 					return r[0]; 				} 			} 			return null; 		}; 	}; 	 	/** 	 * Converts the specified string value into its JavaScript Date equivalent using the specified format {String} or formats {Array} and the CultureInfo specific format information. 	 * The format of the string value must match one of the supplied formats exactly. 	 *  	 * Example 	<pre><code> 	// 15-Oct-2004 	var d1 = Date.parseExact("10/15/2004", "M/d/yyyy");   	// 15-Oct-2004 	var d1 = Date.parse("15-Oct-2004", "M-ddd-yyyy");   	// 15-Oct-2004 	var d1 = Date.parse("2004.10.15", "yyyy.MM.dd");   	// Multiple formats 	var d1 = Date.parseExact("10/15/2004", ["M/d/yyyy", "MMMM d, yyyy"]); 	</code></pre> 	 * 	 * @param {String}   The string value to convert into a Date object [Required]. 	 * @param {Object}   The expected format {String} or an array of expected formats {Array} of the date string [Required]. 	 * @return {Date}    A Date object or null if the string cannot be converted into a Date. 	 */ 	$D.parseExact = function (s, fx) { 		return $D.getParseFunction(fx)(s); 	}; }());   ``` |
```

Generated by istanbul at Sun Nov 16 2014 12:08:33 GMT-0800 (PST)
